# Isolation and Differentiation of Adipose-Derived Stem Cells into Odontoblast-Like Cells: A Preliminary *In Vitro* Study

**DOI:** 10.22074/cellj.2021.7325

**Published:** 2021-07-17

**Authors:** Saber Khazaei, Abbasali Khademi, Mohammad Hossein Nasr Esfahani, Mozafar Khazaei, Mohammad Hossein Nekoofar, Paul M. H. Dummer

**Affiliations:** 1. Department of Endodontics, School of Dentistry and Dental Research Centre, Dental Research Institute, Isfahan University of Medical Sciences, Isfahan, Iran; 2. Department of Endodontics, School of Dentistry, Kermanshah University of Medical Sciences, Kermanshah, Iran; 3. Department of Animal Biotechnology, Reproductive Biomedicine Research Center, Royan Institute for Biotechnology, ACECR, Isfahan, Iran; 4. Fertility and Infertility Research Centre, Health Technology Institute, Kermanshah University of Medical Sciences, Kermanshah, Iran; 5. Department of Endodontics, School of Dentistry, Tehran University of Medical Sciences, Tehran, Iran; 6. School of Dentistry, College of Biomedical and Life Sciences, Cardiff University, Cardiff, UK

**Keywords:** Mesenchymal Stem Cell, Odontoblast, Regenerative Endodontics

## Abstract

**Objective:**

The aim of present study was to isolate and differentiate human adipose-derived stem cells (ASCs) into odontoblast-like cells.

**Materials and Methods:**

In this experimental study, human adipose tissues were taken from the buccal fat pad of three individuals (mean age: 24.6 ± 2.1 years). The tissues were transferred to a laboratory in a sterile culture medium, divided into small pieces and digested by collagenase I (2 mg/mL, 60-90 minutes). ASCs were isolated by passing the cell suspension through cell strainers (70 and 40 µm), followed by incubation at 37ºC and 5% CO2in Dulbecco’s modified eagle medium (DMEM) supplemented with fetal bovine serum (FBS 5%) and penicillin/streptomycin (P/S). After three passages, the ASCs were harvested. Subsequently, flow cytometry and reverse transcriptase polymerase chain reaction (RT-PCR) were used to detect expression levels of NANOG and OCT4 to evaluate stemness. Then, a differentiation medium that included high-glucose DMEM supplemented with 10% FBS, dexamethasone (10 nM), sodium β-glycerophosphate (5 mM) and ascorbic acid (100 µM) was added. The cells were cultivated for four weeks, and the odontogenic medium was changed every two days. Cell differentiation was evaluated with Alizarin red staining and expressions of collagen I (COL1A1), dentin sialophosphoprotein (DSPP) and dentin matrix protein-1 (DMP1).

**Results:**

The ASCs were effectively and easily isolated. They were negative for CD45 and positive for the CD105 and CD73 markers. The ASCs expressed OCT4 and NANOG. Differentiated cells highly expressed DSPP, COL1A1 and DMP1. Alizarin red staining revealed a positive reaction for calcium deposition.

**Conclusion:**

ASCs were isolated successfully in high numbers from the buccal fat pad of human volunteers and were differentiated into odontoblast-like cells. These ASCs could be considered a new source of cells for use in regenerative endodontic treatments.

## Introduction

Stem cells (SCs) have self-renewal ability and the potential to differentiate into several kinds of mature cells, including cardiac, nerve and cartilage; they also maintain their survival and do not undergo atrophy and premature hyperplasia (1). In general, SCs are divided into two groups - embryonic and postnatal, depending on their characteristics. Embryonic SCs (ESCs) have a great ability to differentiate, but their application is associated with substantial medical ethics challenges. Postnatal SCs are undifferentiated cells that are located among differentiated and specialized cells of various tissues (2).

A type of postnatal SC, described as non-hematopoietic SCs that reside in the bone marrow is called mesenchymal SCs (MSCs). These cells are multipotent and can be isolated from several tissues without serious ethical problems; they can also be multiplied *in vitro* (3). Importantly, MSCs derived from various tissues, although having similar general properties, are not exactly alike and vary in terms of proliferation and immune suppression capacity and ability to differentiate into different tissues (4). 

Several types of progenitor/SCs specific to dental tissues have been isolated and identified (5). Moreover, there are other oral tissues from which MSCs can be isolated (6, 7). The use of host SCs reduces inflammatory responses and potential problems with cross infection; therefore, progenitor and SCs of adult tissues such as dental pulp SCs (DPSCs) (8), SCs from human exfoliated deciduous teeth (SHEDs) (9), SCs of the apical papilla (SCAPs) (10) and bone marrow SCs (BMSCs) (11) have been used to regenerate pulp tissue. There are various reports that discuss transplantation of MSCs from dental tissue into root canals for endodontic regeneration, and many protocols have been suggested that use the cell-based approach (12, 13). Nevertheless, for most adult patients with a necrotic tooth who are candidates for pulp regeneration, the majority of the MSCs from dental tissues, including DPSCs, SHEDs and SCAPs, are not available. This might open up a new idea of using other sources of MSCs. At the same time, there is a move towards the use of adipose-derived SCs (ASCs) in regenerative medicine (14, 15).

ASCs can be extracted in large volumes and have the capability to grow and proliferate in great numbers. In addition, the efficacy of ASCs, unlike other MSCs (16), does not change with age, and is not affected by gender, obesity and various diseases, such as vascular diseases (17). Several studies have reported that ASCs generate the nerve growth factors that improve remyelination in impaired nerves and are more resistant to apoptosis (18, 19). ASCs can also express specific characteristics of nerve and glial cells (20).

Given the advantages and appropriateness of this available resource of SCs, the present study aimed to assess the isolation and differentiation of ASCs into odontoblast-like cells. 

## Materials and Methods

The protocol of the present experimental study was approved by the Regional Bioethics Committee affiliated with Kermanshah University of Medical Sciences (KUMS), Kermanshah, Iran (#3009137 and #IR.KUMS. REC.1398.862).

### Isolation of adipose-derived stem cells

Human adipose tissue was taken from additional unwanted fat from the buccal fat pad of three patients (2 females and 1 male, following their informed consent) who were candidates for maxillary LeFort osteotomies. Their mean age was 24.6 ± 2.1 years. The samples were transferred to a laboratory under sterile conditions using a culture medium. The tissues were chopped and digested by collagenase I (Sigma-Aldrich, Germany, 2 mg/mL, 60- 90 minutes). The cell suspension was then centrifuged for 10 minutes at 1500 rpm. Then, the ASCs were isolated by passing the cell suspension through cell strainers (70 and 40 µm), and the ASCs were incubated at 37ºC and 5% CO2 in Dulbecco’s modified eagle medium (DMEM, Gibco, Germany) supplemented with 5% fetal bovine serum (FBS, Gibco) plus penicillin/streptomycin (P/S, Gibco, Denmark).

### Flow cytometry

Mesenchymal (CD105, CD73) and non-mesenchymal (CD45) markers were used to confirm the stemness of the ASCs. Passage-3 isolated ASCs were washed twice with flow cytometry buffer that contained phosphate-buffered saline (PBS) plus 0.5% bovine serum albumin. Anti-CD105-PE, anti-CD73-PreCP and anti-CD45-FITC were used for identification of the ASCs. The cells were incubated with 10 μL of each isotype antibody for 45 minutes at 4˚C. The isolated ASCs were washed three times with flow cytometry buffer and fixed with 1% paraformaldehyde.

### Differentiation of adipose-derived stem cells

The ASCs were cultured in differentiation medium that contained high-glucose DMEM supplemented with FBS (10%), dexamethasone (10 nM, Sigma-Aldrich, Germany), sodium β-glycerophosphate (5 mM, Sigma-Aldrich, Germany) and ascorbic acid (100 µM, Sigma-Aldrich, Germany) for 4 weeks (Table 1) (21, 22)

**Table 1:** Differentiation protocol


Material^*^	Company	Concentration

Dexamethasone	Sigma-Aldrich	10 nM
Sodium β-glycerophosphate	Sigma-Aldrich	5 mM
Ascorbic acid	Sigma-Aldrich	100 µM


* ; Differentiation medium consisted of the above materials added to high-glucose Dulbecco’s modified eagle medium (DMEM) supplemented with 10% fetal bovine serum (FBS).

### Reverse transcriptase polymerase chain reaction 

After three passages and to ensure that no false positive response was present for expressions of NANOG and OCT4, we compared the ASCs to precharacterised SHEDs (23) in terms of expression levels of NANOG and OCT4 for evaluation of stemness. To analyse differentiation, dentin sialophosphoprotein (*DSPP*), dentin matrix protein (*DMP*) and collagen I (*COL1A1*) gene expressions by using a semi-quantitative polymerase chain reaction (PCR) were performed. One μL of cDNA was used as a template in reverse transcriptase PCR (RT-PCR) and was added to 12.5 μL of 2x Master Mix RED (1.5 mM MgCl_2_, Merck, Germany) that included 150 mM Tris-HCl (pH=8.5), 40 mM NH_4_ , 3 mM MgCl_2_ , 0.2% Tween® 20, 0.4 mM of each dNTP, 0.2 U/μL Amplicon Taq DNA polymerase, an inert red dye and stabilizer, 1 μL of each primer (10 μM), and up to 25 μL nuclease-free water. Amplification was carried out using a thermocycler (Eppendorf AG 22331, Hamburg, Germany). The conditions for RT-PCR amplification were as follows: an initial denaturation at 94˚C for 5 minutes, 30 cycles of three-step PCR that consisted of 94˚C for 20 seconds, 60˚C for 25 seconds and 72˚C for 45 seconds, and a final extension at 72˚C for 10 minutes. The RT-PCR output was used for the electrophoresis agarose gel (1.5%) along with molecular weight markers. Table 2 lists the primers used in this study.

### Mineralization evaluation 

The cells were incubated with 40 mM Alizarin red stain (pH=4.2) for 10 minutes. Then, the cells were washed five times with PBS. After each wash, the cells were centrifuged with PBS to reduce the non-specific Alizarin red stain dye and were analysed to detect calcified nodular deposition.

**Table 2:** Gene primer sequences


Gene	Accession number	Annealing temperature (˚C)	Size (bp)	Primer sequence (5ˊ-3ˊ)	Reference

DSPP	NM_014208	60	118	F: CAGTACAGGATGAGTTAAATGCCAGTG	(24)
				R: CCATTCCCTTCTCCCTTGTGACC	
DMP1	NM_004407	60	211	F: GAGAGTCAGAGCGAGGAA	Present study
				R: CTTGGCAGTCATTGTCATC	
COL1A1	NM_000088	60	128	F: GTGCTAAAGGTGCCAATGGT	(25)
				R: ACCAGGTTCACCGCTGTTAC	
NANOG	NM_024865	60	158	F: CAAAGGCAAACAACCCACTT	(26)
				R: TCTGCTGGAGGCTGAGGTAT	
POU5F1 (OCT-4)	NM_002701	60	110	F: AGTGAGAGGCAACCTGGAGA	(27)
				R: ACACTCGGACCACATCCTTC	


## Results

**Fig.1:**
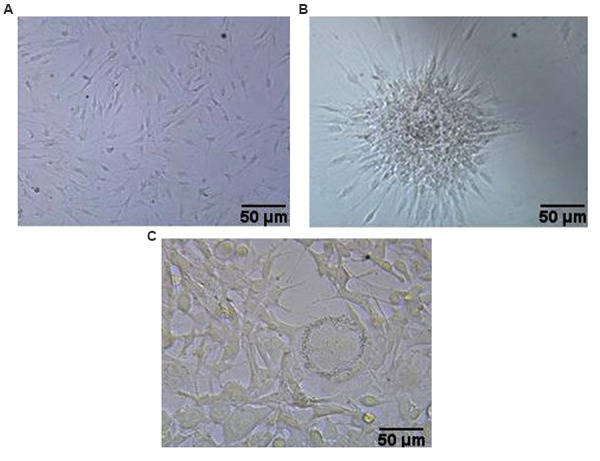
*In vitro *culture of isolated adipose-derived stem cells (ASCs) during different culture periods. **A.** After one week, **B. **Cellular sphere at passage three, and **C. **Odontoblast-like cells after 28 days treatment by differentiation medium (scale bar: 50 µm).

After 24 hours of culture, we observed small, spheroid and translucent ASCs. The ASCs were passaged when they became 70-80% confluent; at this stage the ASCs had a spindle morphology (Fig.1). 

**Fig.2:**
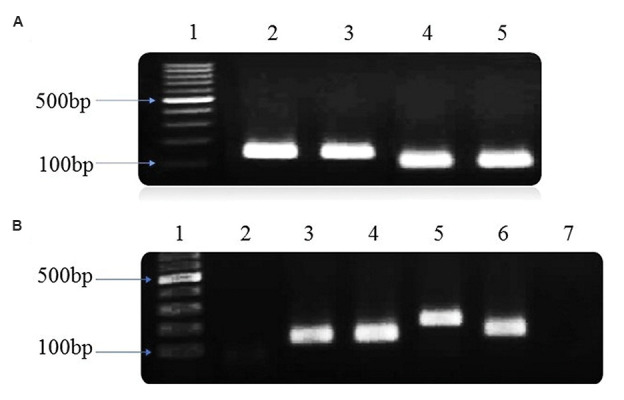
Reverse transcriptase polymerase chain reaction (T-PCR) of stemness genes of adipose-derived stem cells (ASCs) and odonobaslt-like cell specific genes. **A.** Representative example of RT-PCR for gene expression analysis. 1; 100 bp DNA ladder (Lad), 2; *NANOG* (SHEDs), 3; *NANOG* (ASCs), 4; *OCT4* (SHEDs), 5; *OCT4* (ASCs). **B. **Representative example of RT-PCR for gene expression analysis. 1; 100 bp DNA ladder (Lad), 2; *DSPP* after 14 days treatment with differentiation medium, 3; *DSPP* after 21 days treatment with differentiation medium, 4; *DSPP* after 28 days treatment with differentiation medium, 5; *DMP1*, 6; *COL1A1*, 7; Control group (ASCs after 28 days treatment with culture medium without the differentiation medium).

**Fig.3:**
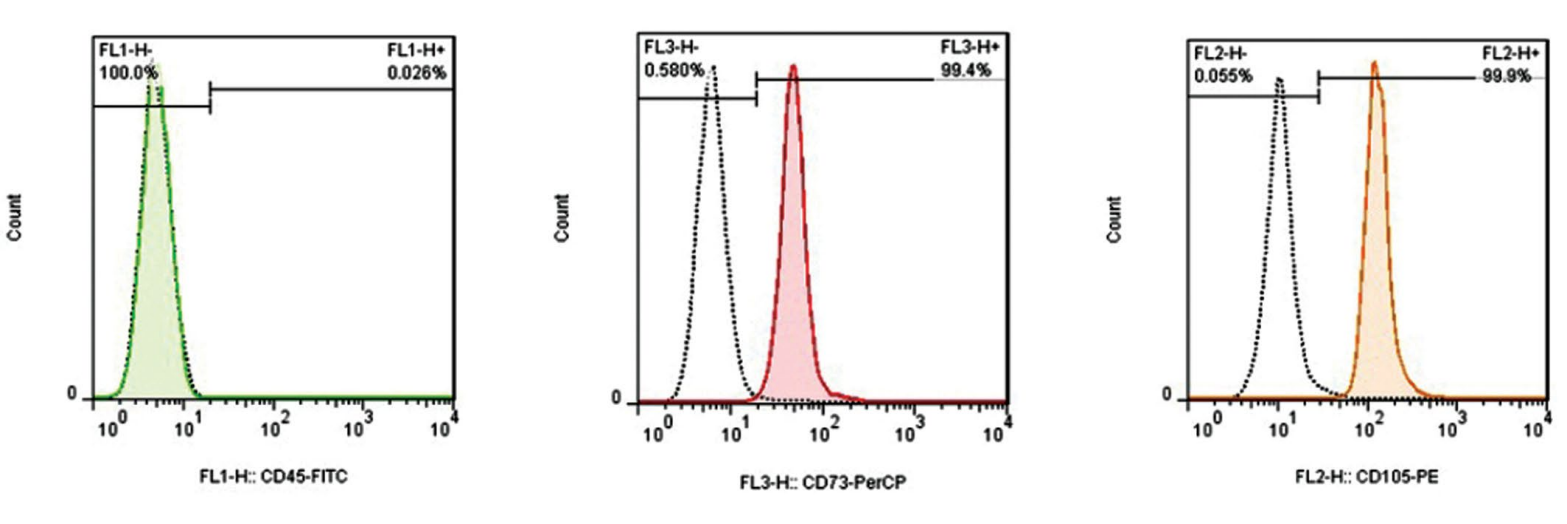
Flow cytometry results show that the isolated adipose-derived stem cells (ASCs) were positive for CD105, CD73, and negative for CD45.

At passage three, the ASCs expressed OCT4 and NANOG (Fig.2), similar to precharacterised SHEDs. This showed that there was no false-positive response to the expressions of NANOG and OCT4. The flow cytometry results showed that isolated the ASCs were positive for CD105 and CD73, and negative for CD45 (Fig.3).

The ASCs differentiated into odontoblast-like cells after four weeks incubation in differentiation medium. The cells increased in size, and became nodular with a more oval and/or round shape (Fig.1C). Expressions of three genes associated with odontoblast-like cell differentiation *(COL1A1, DSPP,* and *DMP1* genes) were confirmed (Fig.2).

**Fig.4:**
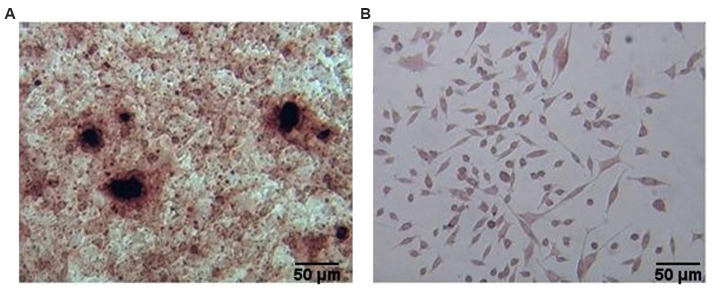
Alizarin Red Stainaning of odontoblast-like cells. **A.** Calcium deposition of odontoblast-like cells using Alizarin red staining after 28 days of treatment with differentiation medium. **B. **Control group (scale bar: 50 µm).

ASCs that differentiated into odontoblast-like cells after four weeks were analysed for mineralization with Alizarin red staining. There was detectable calcified nodular deposition in these ASCs compared with the control group (Fig.4).

## Discussion

Several types of SCs specific to dental tissues (DPSCs, SCAPs, SHEDs and BMSCs) have been isolated and evaluated for endodontic regeneration (8-11). MSCs that are derived from various tissues are similar in terms of general characteristics; however, they differ in terms of proliferation, immune suppression, and the ability to differentiate into various tissues (7). To date, no study has evaluated the differentiation potential of ASCs from human buccal fat pad into odontoblast-like cells. It has been reported that this adipose tissue may have neural crest origin (28), and the present study supports the concept that differentiation into odontogenic-like cells can occur in ASCs from the human buccal fat pad if sufficient signals are provided.

ASCs have self-renewal ability and potential to differentiate into various lineages of mesenchymal tissue. These cells resemble similar surface antigens such as MSCs, but are not identical to BMSCs (29). *In vitro* studies have shown that they can differentiate into different lineages, including adipocytes, cartilage, bone, muscle, hematopoietic, neural, liver, angiogenic, and epithelial cells (30). ASCs express mesenchymal markers such as CD90, CD44 and CD105, and negative expression of the hematopoietic markers CD14, CD34 and CD45 (31). The results of the present study revealed that ASCs expressed CD90, CD105, OCT4 and NANOG, as do SHEDs.

Several protocols and growth factors have been introduced for differentiation of MSCs into odontoblast-like cells. These include bone morphogenetic proteins (BMPs) (32), transforming growth factor (TGFβ1-3) (32), nerve growth factor (NGF) (33) and fibroblast growth factor (FGF-2) (34). Wu et al. (35) reported that ASCs could differentiate into odontoblast-like cells using the inguinal fat pads of mice as a source for ASCs. In the present study, we used a simple and inexpensive protocol that was composed of dexamethasone, sodium β-glycerophosphate and ascorbic acid. By altering the composition of these growth factors, the differentiation of these cells was altered and the cells had the capability to express markers of odontoblasts or osteoblasts, depending on their exposure to different combinations of growth factors. Other studies have evaluated growth factors administered alone or in different combinations to enhance differentiation of odontoblast-like cells (36-38).

The results of the present study revealed that these ASCs differentiated into odontoblast-like cells and expressed *COL1A1, DMP1* and *DSPP* genes after four weeks of treatment, whereas after two weeks the cells did not express these markers. DSPP is produced by odontoblasts inside the tooth pulp. However, osteoblasts can also produce this protein. DSPP plays a pivotal role in mineral deposition during dentinogenesis (39). Type I collagen is the major protein of dentin and regulates the expression level of DMP1. It has been demonstrated that type I collagen and DMP1are expressed mainly in active odontoblasts (40).

It has been reported that ASCs could be used for regeneration (15). However, human ASCs, particularly buccal fat pad for complete pulp regeneration, has not been evaluated. Using this line of cells may open new fields of research for endodontic regeneration treatments. They can be extracted in large volumes from the buccal fat pad and can grow and proliferate in large numbers. In addition, the efficacy of ASCs, unlike other MSCs, does not change due to age, gender, or obesity.

## Conclusion

In the present *in vitro* study, high numbers of ASCs were isolated successfully from the human buccal fat pad and were differentiated into odontoblast-like cells. A subsequent *in vivo* study is suggested to evaluate the differentiation potential of ASCs into odontoblast-like cells.
